# Metabolomic Profiling of Different *Antrodia cinnamomea* Phenotypes

**DOI:** 10.3390/jof9010097

**Published:** 2023-01-10

**Authors:** Chun-Han Su, Yun-Cheng Hsieh, Jin-Yi Chng, Ming-Nan Lai, Lean-Teik Ng

**Affiliations:** 1 Department of Food Science, Fu Jen Catholic University, New Taipei City 242062, Taiwan; 2 Department of Agricultural Chemistry, National Taiwan University, No. 1, Sec. 4, Roosevelt Road, Taipei 10617, Taiwan; 3 Kang Jian Biotech Co., Ltd., Nantou 54245, Taiwan

**Keywords:** *Antrodia cinnamomea*, phenotypes, fruiting bodies, metabolomics, triterpenoids

## Abstract

*Antrodia cinnamomea* (AC) is a precious medicinal fungus with numerous therapeutic benefits. Based on the color appearance of its fruiting bodies, AC can be divided into red AC (RAC), yellow AC (YAC), and white AC (WAC); however, the differences in their metabolomic profiles remain unknown. This study aimed to analyze the metabolomic profiles of three different AC phenotypes and examine their relationship to the color appearance of fruiting bodies. The results showed that although RAC, YAC, and WAC appear to have a relatively similar profile of index triterpenoids, their total triterpenoid contents were significantly different. Among the annotated triterpenoids, many of them were highly present in RAC but not in YAC and WAC, and the relative contents of the four ergostanes (antcamphin F, antcamphin L, antcin B, and antcin K) and one lanostane (versisponic acid D) were found to be significantly different among AC phenotypes. The metabolomic profiles of the AC fruiting bodies demonstrated a total of 140 metabolites, and 41 of them were very different among AC phenotypes. This study indicates that red, yellow, and white AC can biosynthesize the diverse structures of triterpenoids, and RAC possesses a relatively higher contents of triterpenoids and diverse unannotated metabolites than YAC and WAC.

## 1. Introduction

*Antrodia cinnamomea* (AC, also known as *Antrodia camphorata*, *Taiwanofungus camphoratus*, and Niu-chang-chih), belonging to the Polyporaceae family, is a rare and precious medicinal mushroom indigenous to Taiwan [1,2]. Traditionally, it is used as a folk medicine to prevent and treat various diseases, including liver disease, cancer, inflammation, and hypertension. Triterpenoids, steroids, benzoquinones, maleic and succinic derivatives, nucleotides, and fatty acids are compounds commonly found in AC fruiting bodies and mycelia. Studies have demonstrated that the fruiting bodies of AC contain a complicated array of tetracyclic triterpenoids, including ergostanes and lanostanes [2,3], as the major bioactive constituents, which were shown to possess anti-cancer, anti-inflammatory, and hepatoprotective activities [2,4,5]. Among the bioactive triterpenoids, antcin A possessed anti-inflammatory activity [6]. Antcin B and methylantcinate B were able to cause extrinsic and intrinsic apoptosis in hepatocellular carcinoma cells [7]. Antcin H exhibited a hepatoprotective effect [8]. Dehydroeburicoic acid and eburicoic acid possess potent anti-diabetic and anti-hyperlipidemic activities [9,10]. Antrocin was found to inhibit the growth of human lung cancer cells [11]. These triterpenoids have relatively similar structures, only differing in the number, location, or stereochemistry of the hydroxyl groups.

Liquid chromatography or gas chromatography coupled with mass spectrometry (LC-MS or GC-MS) have been successfully used to determine the metabolite profile of various plants such as tomato, *Arabidopsis*, date, and potato [12,13,14]; these techniques have been applied to provide very useful information for a better understanding of the genotypic or phenotypic differences in plant species. The fruiting bodies of AC generally appear in red-orange, while yellow and white variants are rarely found in the natural environment. The fruiting bodies of the yellow and white variants are difficult to obtain, so information on their morphology, biology, and chemistry remains limited. Given the increasing demand and the scarcity of AC in the wild, comparative studies on the familiar form (red), yellow, and white variants of AC are essential for selecting elite-quality isolates for a mass cultivation.

The bioactive ingredient content is essential when choosing the type of AC strain to cultivate. To explore the diversity of the metabolites in AC fruiting bodies and their selection for a future mass production, this study conducted a comparative examination of the metabolic profiles of different AC phenotypes and their relationship to the color appearance of fruiting bodies. This information will be beneficial for developing strategies for an elite AC selection and cultivation in the future.

## 2. Materials and Methods

### 2.1. Materials

**Figure 1:**
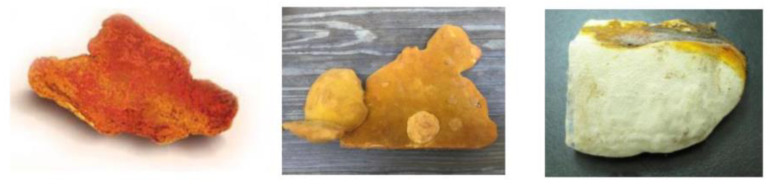
The appearance of fruiting bodies of different *A. cinnamomea* phenotypes. The images from left to right are red AC, yellow AC, and white AC.

Three different *Antrodia cinnamomea* (AC) phenotypes [namely fruiting bodies with red (RAC), yellow (YAC), and white (WAC) color], with each phenotype containing four samples of different strains, which were collected from various regions of Taiwan (Figure 1). The authenticity of all samples was confirmed by the Internal Transcribed Spacer (ITS) Sequence Analysis, followed by comparing their rDNA sequence with the nucleic acid sequence registered in the NCBI database (GenBank). All chemicals used in the metabolite analysis were of an analytical grade.

### 2.2. Determination of Total Triterpenoid Contents

The total triterpenoid content was measured by the colorimetric method as described by Cui et al. [15] with a slight modification. In brief, 20 mg of AC fruiting body powder were taken and mixed with 1 mL of 80% ethanol, followed by extracting under ultrasonic shaking for 15 min. After centrifugation at 10,000× *g* for 5 min, the supernatant was collected and made up to a volume of 10 mL with 80% ethanol, of which 1 mL was taken and filtered through a 0.45 μm polytetrafluoroethylene (PTFE) membrane filter and then evaporated to dryness, followed by adding 0.4 mL of 5% vanillin-glacial acetic acid solution and 1 mL of perchloric acid. The sample was heated for 20 min in a 60 °C water bath. After cooling with ice water, 10 mL of glacial acetic acid was added, and the sample was shaken for 15 min at room temperature. The sample absorbance was measured at a wavelength of 550 nm. The total triterpenoid content was determined using the standard oleanolic acid calibration curve and expressed as oleanolic acid equivalents (OAE, mg/g dry weight).

### 2.3. Analysis of AC Index Compounds

The analysis of the characteristic compounds of AC was performed according to the method of Lin et al. [4]. In brief, 200 mg of the sample were taken and placed in a 15 mL tube, followed by adding 0.8 mL of 95% ethanol. After sonication in an ultrasonic bath at room temperature for 1 h, the sample was centrifuged at 6,000× *g* for 5 min. The supernatant was taken and filtered with a 0.22 μm PTFE membrane filter. Alcohol (95%) was added to the filtrate to a constant volume of 1 mL, and then the sample was stored at −20 °C until the analysis.

The high-performance liquid chromatography (HPLC) was performed using an Agilent 1100 system (Agilent Technologies Inc., Santa Clara, CA, USA) comprising a G1311A quaternary pump and a UV–visible spectroscopic detector (Agilent model G1314A) set at a wavelength of 254 nm for the detection. Chromatographic separation was performed on a Luna C18(2) column (5 µm, 4.6 × 250 mm, Phenomenex Inc., Torrance, CA, USA). The mobile phase consisted of A: H_2_O (containing 0.1% acetic acid), B: methanol, and C: acetonitrile. The flow rate was set at 0.5 mL/min between 0 and 95 min and 1.0 mL/min between 95 and 115 min. The elution gradient at 0 min was 40% A, 30% B, and 30% C; at 5 min, 40% A, 30% B, and 30% C; at 95 min, 10% A, 10% B and 80% C; 105 min, 0% A, 0% B and 100% C; and at 115 min, 0% A, 0% B, and 100% C. The sample injection volume was 20 µL. The peak area was calculated by Agilent OpenLab ChemStation software. The bioactive constituents were identified by comparing their relative retention times with the published data in the literature.

### 2.4. Metabolomic Analysis

Based on the procedures described by Qiao et al. [3], all analyses were performed by an Agilent 1260 Infinity LC system (Agilent Technologies Inc., Santa Clara, CA, USA) connected to a mass spectrometer (Orbitrap Elite-ETD, Thermo Fisher Scientific Inc., Waltham, MA, USA). Chromatographic separation was performed using an ACQUITY UPLC BEH C18 column (1.7 µm, 2.1 mm × 50 mm, Waters, Milford, MA, USA). The mobile phase comprised A: H_2_O (containing 0.1% acetic acid) and B: acetonitrile. The flow rate was 0.3 mL/min. The mobile phase gradient was: 0 min, 30% B; 3–5 min, 53% B; 12 min, 90% B; and 15–18 min, 95% B. The injection volume of the sample was 5 μL. The measurement was conducted in negative electrospray ionization (ESI) mode. High-purity nitrogen was used as the sheath gas at 30 psi and as the auxiliary gas at 10 psi. High-purity argon was used as the collision gas at 1.5 mTorr. The spray voltage of the ion source was 4 kV, and the capillary temperature was 320 °C. The scanning range was set as *m*/*z* 100–1000 with an accumulation time of 0.10 s.

The liquid chromatography-tandem mass spectrometry (LC-MS/MS) data processing, including the peak extraction, alignment, and creation of the data matrix, was performed using MZmine software (http://mzmine.sourceforge.net, accessed on 4 January 2023), and a metabolite identification was carried out as previously described [3]. The peak areas were obtained using the automatic integration function provided by the MZmine software. SIRIUS software was used to classify and predict the compound’s structure [16].

### 2.5. Statistical Analysis

All values are expressed as the mean ± standard deviation. The differences between the means of the groups were evaluated by the analysis of variance (ANOVA) and then tested by the Least Square Difference (LSD). *p* < 0.05 was considered statistically significant.

LC-MS data matrices, including the relative abundance of each signal (metabolite) in each sample, were submitted for unsupervised (principal component analysis, PCA) and supervised multivariate statistical analyses (orthogonal partial least squares-discriminant analysis, OPLS-DA) using SIMCA 13.0 (Umetrics) as previously described [17]. The online MetaboAnalyst (https://www.metaboanalyst.ca, accessed on 4 January 2023) statistical tool and R statistical software were used to analyze the data.

## 3. Results and Discussion

### 3.1. Authentication of A. cinnamomea Phenotypes

After aligning the ITS sequences of the different phenotypes of AC with the standard AC gene sequences in the NCBI gene database, the results showed that the AC strains of the three phenotypes collected from various regions in Taiwan were shown to have an almost 100% similarity in the ITS sequences, confirming that they were AC. The information of these ITS sequences has been registered in the NCBI GenBank database with the ID numbers MK764936, MN947413, MN947414, and MN947415 for red AC; MK764937, MN947416, and two remain to be registered ITS sequences for yellow AC; and MK764938, MN947417, MN947418, and MN947419 for white AC (Table S1).

### 3.2. Triterpenoid Contents and Profiles of Different A. cinnamomea Phenotypes

**Figure 2:**
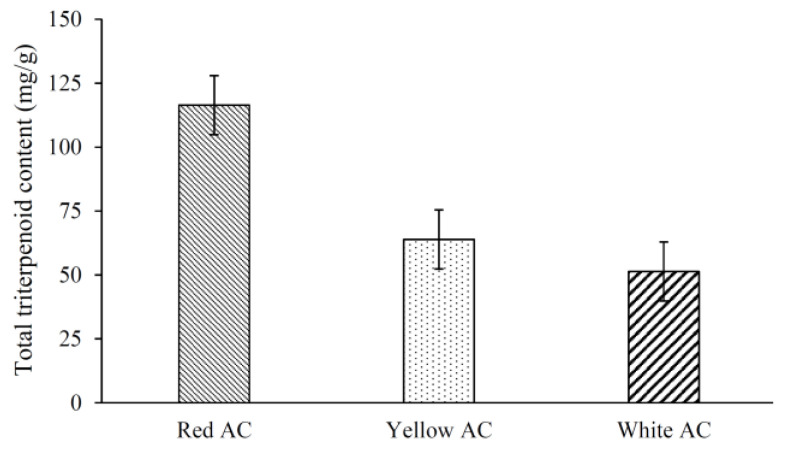
Total triterpenoid contents in fruiting bodies of different *A. cinnamomea* phenotypes. Values are mean ± SD (*n* = 4).

AC fruiting bodies contain diverse and abundant triterpenoids, which possess good anti-tumor, anti-inflammatory, hepatoprotective, and other beneficial effects [1,2,3]. The results showed that the total triterpenoid contents of different AC phenotypes were significantly different (Figure 2), with RAC (116.4 mg/g) having the highest content, followed by YAC (63.9 mg/g), and the lowest was in WAC (51.3 mg/g).

**Figure 3:**
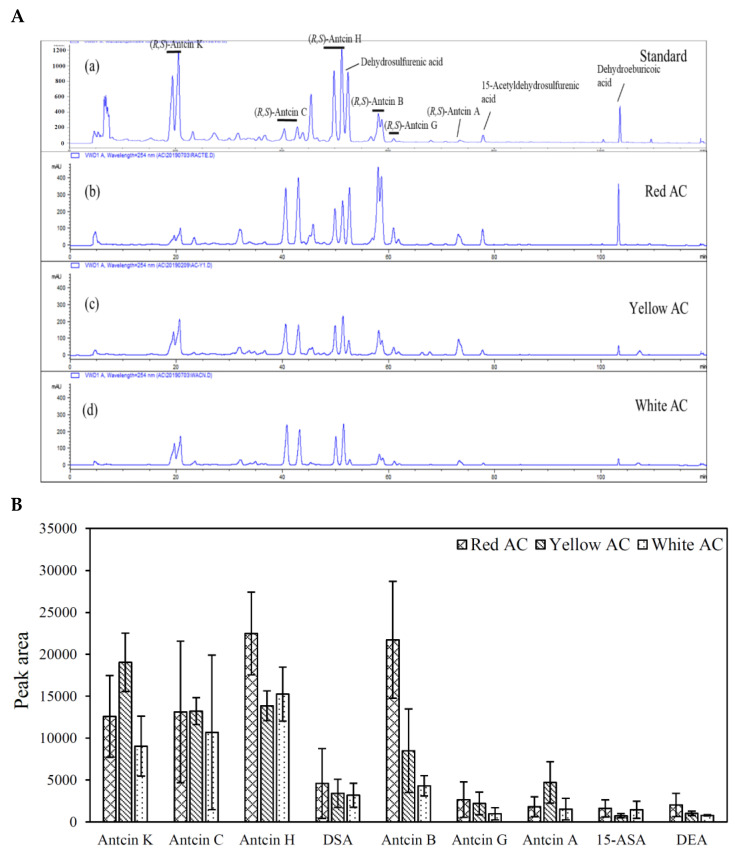
Representative HPLC profiles and peak areas of bioactive triterpenoid (index) compounds in fruiting bodies of different *A. cinnamomea* phenotypes collected from the wild. (**A)**. Representative HPLC profiles: (**a**) standard, (**b**) red AC, (**c**) yellow AC, and (**d**) white AC. (**B**). The peak area represents the concentration of the compound integrated and calculated automatically by the computer of the HPLC system. DSA = Dehydrosulfurenic acid; 15-ASA = 15-Acetyldehydrosulfurenic acid; DEA = Dehydroeburicoic acid.

The structures of the AC triterpenoids mainly possess an ergostane skeleton (e.g., antcins A, C, K, and zhankuic acids A, B, and C) and a lanostane skeleton (e.g., sulfurenic acid, dehydrosulfurenic acid, eburicoic acid, and dehydroeburicoic acid). According to Lin et al. [4] and CNS [18], eight of the identified triterpenoids, including antcin A, antcin B, antcin C, antcin H, antcin K, dehydrosulfurenic acid, 15-acetyldehydrosulfurenic acid, and dehydroeburicoic acid, were recommended as index components for the quality control of the raw materials and products of the AC fruiting bodies. Figure 3A shows the chromatographic profiles of the AC fruiting bodies of different phenotypes. The results showed that RAC, YAC, and WAC appear to have a relatively similar profile index of the triterpenoid compounds; however, their relative contents were different (Figure 3B). RAC appears to have higher relative contents in antcin B, antcin C, antcin G, antcin H, dehydrosulfurenic acid, 15-acetyldehydrosulfurenic acid, and dehydroeburicoic acid than the YAC and WAC phenotypes; however, its relative contents of antcin A and K were lower than YAC phenotype. When the relative contents of each triterpenoid were added up, RAC was shown to have the highest total amount of these index compounds, followed by YAC, and the lowest was in WAC. This observation was consistent with the distribution of the total triterpenoids in different phenotypes, indicating that the total triterpenoids of different AC phenotypes are mainly composed of the triterpenoid index components.

Previous studies have pointed out that white and red AC have similar index components, including antcin A, antcin B, antcin C, antcin H, antcin K, dehydrosulfurenic acid, dehydroeburicoic acid, and 1,4-dimethoxy-2,3-methylenedioxy-5-methylbenzene (non-triterpenoid) [19]. In addition, consistent with previous studies, the content of total triterpenoids in the fruiting bodies of RAC was higher than WAC [19,20], while there is no literature report on the total triterpenoid content of YAC.

### 3.3. Untargeted Metabolomic Analysis of Different A. cinnamomea Phenotypes

**Figure 4:**
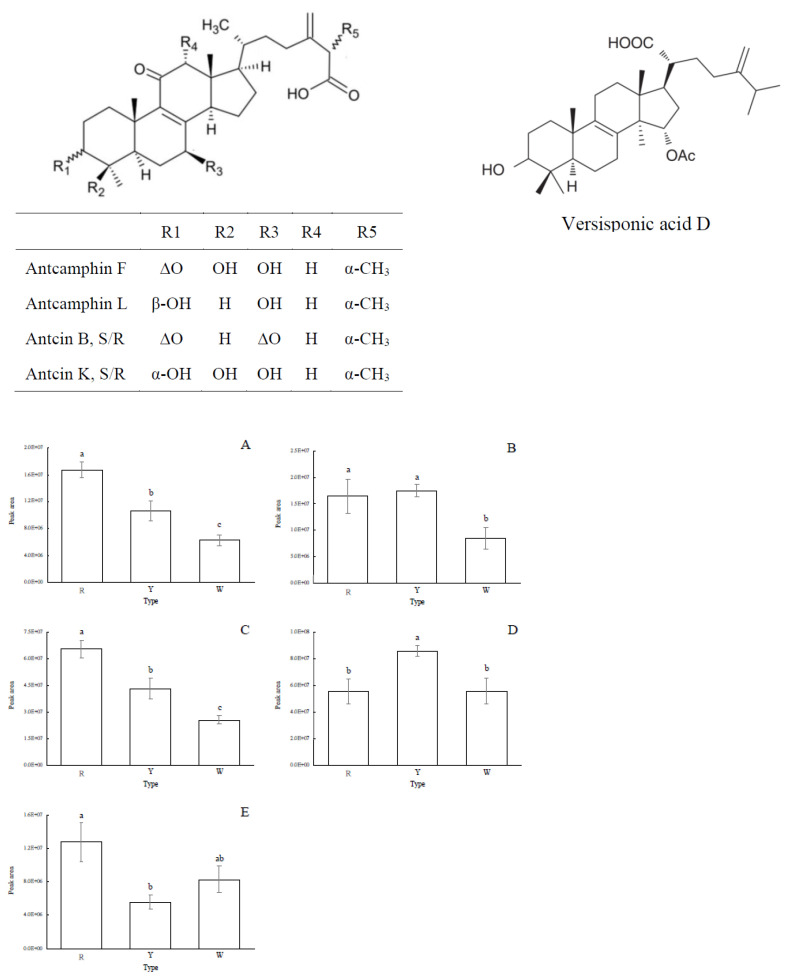
Differences in the relative content of the compounds with known structure in different *A. cinnamomea* phenotypes. (**A**): antcamphin F, (**B**): antcamphin L, (**C**): antcin B, (**D**): antcin K, and (**E**): versisponic acid D. Values are mean ± SD (*n* = 4). Different letters in the same figure indicate significant differences between different AC phenotypes (*p* < 0.05 as analyzed by LSD tests). R = red AC; Y = yellow AC; W = white AC. The peak area represents the amount of a compound present in the sample as integrated and calculated automatically by the LC-MS chromatographic system.

To dissect the diversity of metabolites between AC phenotypes, we performed a non-targeted metabolome analysis on 12 AC samples of three different phenotypes collected from various regions of Taiwan. The results showed that RAC, YAC, and WAC exhibited apparent differences in their mass spectral signals, demonstrating a total of 140 compounds with *m*/*z* values ranging from 285 to 976. After data pre-processing and comparing with the in-house database and the online databases, a total of 26 triterpenoids were putatively identified (Table 1), of which five of the known bioactive triterpenoids (i.e., antcamphin F, antcamphin L, antcin B, antcin K, and versisponic acid D) exhibited a significant difference between AC phenotypes (Figure 4). These triterpenoid structures have an ergostane skeleton or a lanostane skeleton. It is generally believed that ergostane-type triterpenoids are produced in fruiting bodies, and lanostane-type triterpenoids exist both in fruiting bodies and in mycelia [1].

**Figure 5:**
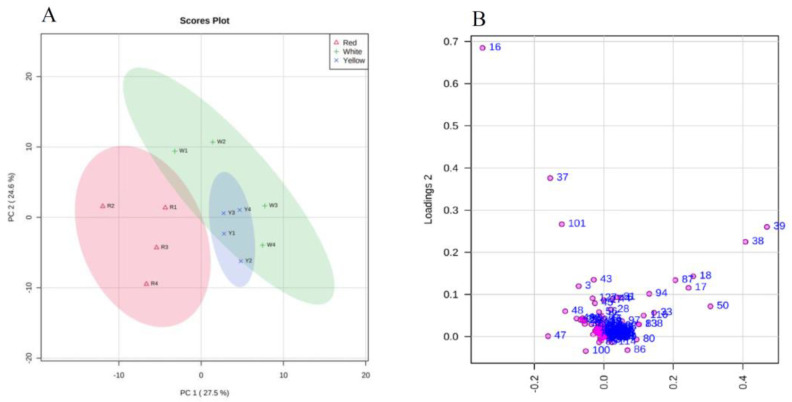
Principal component analysis of fruiting bodies of different *A. cinnamomea* phenotypes. (**A**): Score plot; Component 1 = 27.5%; component 2 = 24.6%. Different color represents different AC phenotypes as indicated in the legend. (**B**): Principal component analysis (PCA)-derived loading plot of the two principal components (PC1 and PC2) of 140 metabolites from different AC phenotypes.

Principal component analysis (PCA) was performed based on the LC-MS/MS data of all samples to evaluate the metabolomic difference among different AC phenotypes. The two-dimensional principal component score plot is composed of the first two principal components (Figure 5A), which shows the clustering trend of the components of AC in different phenotypes; RAC and WAC can be distinguished, while some characteristics of YAC appear to be between RAC and WAC. The PCA diagram shows that the principal components (PC) 1 and 2 can explain 27.5% and 24.6% variability, respectively. The PC1 clearly separated the samples of three AC phenotypes, indicating the diversity of the metabolites between AC phenotypes. According to PC2, the samples of individual AC phenotypes were clustered together. Meanwhile, the clusters of WAC and YAC got close to each other but not close to the cluster of RAC.

The loading plot of the PCA was drawn to explore further the characteristic compounds that have a more significant contribution to the principal components (Figure 5B). It was found that there were nine compounds with a weight greater than 0.2 in PC1 or PC2, namely: 25*S*-antcin K, 25*S*-antcin C, 25*R*-antcin C, 25*S*-antcin B, compound 16, compound 17, compound 18, compound 87, and compound 101 (Table S2). In addition, there are 31 unidentified compounds (the molecular formula is predicted based on calculating the exact mass with an *m*/*z* difference of less than 1 ppm), which are significantly different between the phenotypes (Table S3).

**Figure 6:**
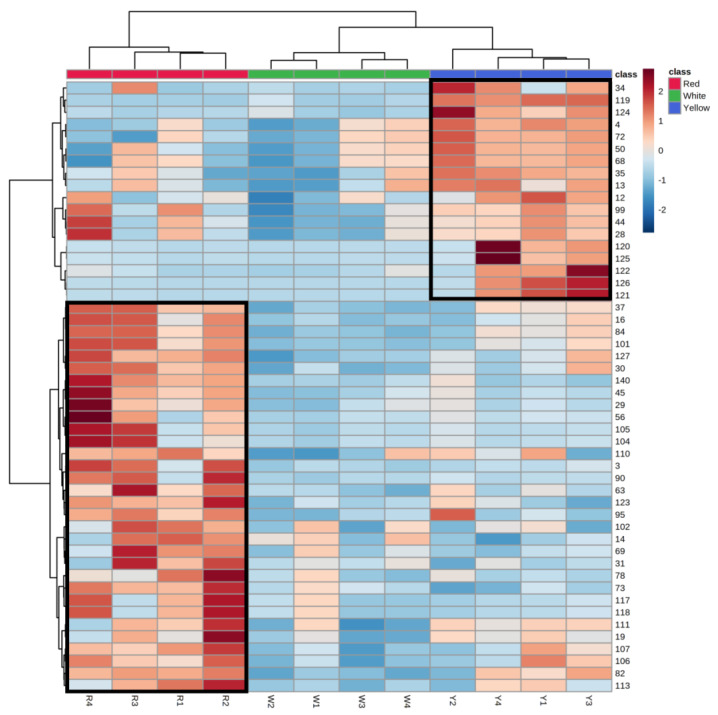
Heatmap of top 50 differential metabolites. The class with a different number of each row represents the compound ID. The class red (R), white (W), and yellow (Y) represent red AC, white AC, and yellow AC, respectively. The relative abundance of metabolites (which ranges from −2 to +2) in different *A. cinnamomea* phenotypes are calculated by the mean-centered and divided by the standard deviation of each variable (metabolites).

The species-dependent accumulation pattern was further visualized by a heatmap based on the metabolome data. Figure 6 shows the heatmap of the top 50 differential metabolites in the different AC phenotypes. The heatmap results showed that RAC has a group of unique metabolites (from the row of ID 37 to the row of ID 113), their contents were different from WAC and YAC, and another group of metabolites (from the row of ID 34 to the row of ID 121) are unique metabolites in YAC. According to the compound classification results obtained by using SIRIUS software [16], these metabolites mainly belong to steroids and steroid derivatives (ID# 34, 35, 13, 12, 28, and 122) and prenol lipids (ID# 72, 50, 68, 99, 44, 120, and 125; metabolite 50 is a bioactive triterpenoid, antcin K, while metabolite 44 is antcamphins L and R). However, metabolites 119, 124, 126, and 121 could not map to a suitable compound classification by the SIRIUS software.

In RAC, unique metabolites include 12 steroids and steroid derivatives, 9 prenol lipids, and 11 metabolites with unannotated classes, while metabolites 37, 127, and 106 were identified to be antcin B, antcamphin F, and antcin C, respectively, and metabolites 45 and 56 are novel ergostanes. Similar to YAC, two RAC samples (R1 and R4) also had high contents of metabolites 99, 44, and 28, while some RAC metabolites (37, 16, 84, 101, 111, 19, 107, 106, 82, and 113) were present in a moderate amount in the YAC, indicating that the pathway genes of these metabolites may exist in both RAC and YAC. The heatmap results also suggest that the metabolic pathway activities of steroids, steroid derivatives, and prenol lipids appear to differ between yellow and red AC fruiting bodies. Although the differences in the contents of significant metabolites in the same color AC fruiting bodies were minimal, the trend of the metabolite content caused by the fruiting body color of AC is undeniable, indicating that there are differences in the metabolomic profiles among the AC phenotypes; these metabolites may be directly related to the color of the AC fruiting bodies. In particular, metabolites 119 and 124 are two major characteristic compounds of YAC; their content is significantly higher than in the fruiting bodies of the other two phenotypes, suggesting that these two metabolites may be related to the yellow appearance. The metabolite 3 is putatively annotated as a retinoid by SIRIUS software, which is a yellow-red color. Its content is closely associated with the color intensity of the AC fruiting bodies, indicating that it could be an essential metabolite that affects the color of the AC phenotypes.

To fulfill the market demand for AC products, many culture techniques, such as a solid-state (i.e., wood-log, plastic bags, or space bags) culture, liquid culture/submerged fermentation, and dish culture, have been developed to mass-produce AC materials [21]. However, differences in the profile of bioactive metabolites were noted between the fruiting bodies and cultured mycelia of AC, and their production techniques [22,23]. In addition, studies also showed that different AC strains have different metabolic activities, chemical profiles, and potency of bioactivities [24].

To date, more than 200 compounds, including polysaccharides, triterpenoids, ubiquinone derivatives, maleic and succinic acid derivatives, benzene derivatives, and glycoprotein, have been isolated from the AC fruiting bodies and mycelia [2,3,25]. Among them, ergostane and lanostane triterpenoids are considered the main bioactive compounds of AC, of which ergostane-type triterpenoids are considered to be the unique bioactive compounds of AC and are only found in its fruiting bodies [1,25].

The alteration in the growth conditions can activate the production of fungal secondary metabolites [26]. Chen et al. [27] reported that blue-light radiation could change the color of the regular AC from orange-red to white by changing its secondary metabolism and growth condition. A culture media containing the bark extract or wood chips of *Cinnamomum kanehirae* [28,29] and extracts from *Cinnamomum camphora* stem [28,29] or citrus peel [30] were shown to significantly enhance the triterpene content in AC mycelia. Furthermore, the amounts of ergostane-type triterpenoids increased, whereas the lanostane-type triterpenoids in fruiting bodies decreased with an increased culture age [4]. The yield of triterpenoids could be increased in AC cocultured with *Saccharomyces cerevisiae* [31]. Using rapid and repeated submerged fermentation, asexual spore inoculation processes were able to significantly improve the production efficiency of the active substances of AC [32]. These results suggest that besides the types of phenotypes, the culture medium and method would also influence the content of triterpenoids in AC fruiting bodies and mycelia.

## 4. Conclusions

This study demonstrated that RAC, YAC, and WAC have a relatively similar profile of index compounds but significantly different amounts of total triterpenoids. The various AC phenotypes were shown to contain different metabolomic profiles. Among the identified triterpenoids with known structures, five triterpenoids were found to present significantly different amounts among AC phenotypes; four are ergostanes (antcamphin F, antcamphin L, antcin B, and antcin K), and the other is versisponic acid D (a lanostane triterpenoid). A total of 140 metabolites were annotated; although the structures of 31 metabolites remain unclear, the relative contents of 41 metabolites were found to be significantly different among the AC phenotypes. RAC was shown to have the highest contents of the most detected metabolites than YAC and WAC. This study also points out that the different AC phenotypes are able to biosynthesize triterpenoids containing carbon atoms between 27 and 30, which may also include triterpenoids with unknown structures.

## Figures and Tables

**Table 1:** Triterpenoid metabolites in different *A. cinnamomea* phenotypes.

				HRMS (*m*/*z*)		
Identity	Triterpenoid Types	Formula	t_R_ (min)	Calculated	Measured	∆*m*/*z* (ppm)
Antcin K, *S* + *R*	Ergostanes	C_29_H_44_O_6_	4.00	487.3065	487.3061	−0.8
Camphoratin A	Ergostanes	C_29_H_44_O_6_	4.31	487.3065	487.3062	−0.6
12-OH antcamphin E/F	Ergostanes	C_29_H_42_O_7_	4.46	501.2858	501.2853	−1.0
12-OH antcamphin E/F	Ergostanes	C_29_H_42_O_7_	4.59	501.2858	501.2852	−1.2
Hydrated antcin F (∆14)	Ergostanes	C_29_H_42_O_6_	4.87	485.2909	485.2906	−0.6
Antcamphin E, *S*	Ergostanes	C_29_H_42_O_6_	5.16	485.2909	485.2905	−0.8
Antcamphin K, *S*	Ergostanes	C_29_H_44_O_5_	5.16	471.3116	471.3113	−0.6
Antcamphin F, *R*	Ergostanes	C_29_H_42_O_6_	5.23	485.2909	485.2904	−1.0
Antcamphin L, *R*	Ergostanes	C_29_H_44_O_5_	5.24	471.3116	471.3113	−0.6
Antcin F, *S* + *R*	Ergostanes	C_29_H_40_O_5_	5.90	467.2803	467.2801	−0.4
Antcin C, *S*	Ergostanes	C_29_H_42_O_5_	6.04	469.2959	469.2957	−0.4
Antcin C, *R*	Ergostanes	C_29_H_42_O_5_	6.25	469.2959	469.2956	−0.6
Antcin H (zhankuic acid C), *R*	Ergostanes	C_29_H_42_O_6_	6.53	485.2909	485.2906	−0.6
Antcin H, *S*	Ergostanes	C_29_H_42_O_6_	6.71	485.2909	485.2905	−0.8
Dehydrosulfurenic acid	Ergostanes	C_31_H_48_O_4_	7.97	483.3480	483.3475	−1.0
Antcin I (zhankuic acid B), *R*	Ergostanes	C_29_H_42_O_5_	8.13	469.2959	469.2957	−0.4
Antcin I, *S*	Ergostanes	C_29_H_42_O_5_	8.15	469.2959	469.2957	−0.4
Sulfurenic acid	Ergostanes	C_31_H_50_O_4_	8.16	485.3636	485.3632	−0.8
Antcin B (zhankuic acid A), *S*	Ergostanes	C_29_H_40_O_5_	8.41	467.2803	467.2800	−0.6
Antcin G (25*R*/*S* epimer)	Ergostanes	C_29_H_44_O_6_	8.78	511.3065	511.3061	−0.8
Antcin A, *S* + *R*	Ergostanes	C_29_H_40_O_4_	10.02	453.3010	453.3008	−0.4
15α-Acetyldehydrosulfurenic acid	Lanostanes	C_33_H_50_O_5_	10.69	525.3585	525.3582	−0.6
Versisponic acid D	Lanostanes	C_33_H_52_O_5_	11.00	527.3742	527.3738	−0.8
3β-Hydroxylanosta-7,9(11), 24(28)-triene-21-oic acid	Lanostanes	C_30_H_46_O_3_	13.12	453.3374	453.3372	−0.4
Dehydroeburicoic acid	Lanostanes	C_31_H_48_O_3_	13.73	467.3531	467.3528	−0.6
Eburicoic acid	Lanostanes	C_31_H_50_O_3_	14.05	469.3687	469.3683	−0.9

Note: HRMS: the mass-to-charge ratio of [M–H]^−^ measured by high-resolution mass spectrometry. *S* and *R* indicate the stereoisomer configuration.

## Data Availability

Not applicable.

## Supplementary Materials

The following supporting information can be downloaded at: https://www.mdpi.com/article/10.3390/jof9010097/s1, Table S1. The phylogenetic identification of different *A. cinnamomea* (AC) phenotypes collected from the wild; Table S2. Compounds having significant contributions to each principal component [greater than 0.2; include principal component 1 (PC1) and principal component 2 (PC2)] in principal component analysis using the scaled relative peak areas (%) as independent variables; Table S3. Significantly different compounds of unidentified structure.

## References

[1] Geethangili M., Tzeng Y.M. (2011). Review of pharmacological effects of *Antrodia camphorata* and its bioactive compounds. Evid. Based Complement. Alternat. Med..

[2] Kuang Y., Li B., Wang Z., Qiao X., Ye M. (2021). Terpenoids from the medicinal mushroom *Antrodia camphorata*: Chemistry and medicinal potential. Nat. Prod. Rep..

[3] Qiao X., Wang Q., Ji S., Huang Y., Liu K.D., Zhang Z.X., Bo T., Tzeng Y.M., Guo D.A., Ye M. (2015). Metabolites identification and multi-component pharmacokinetics of ergostane and lanostane triterpenoids in the anticancer mushroom *Antrodia cinnamomea*. J. Pharm. Biomed. Anal..

[4] Lin T.Y., Chen C.Y., Chien S.C., Hsiao W.W., Chu F.H., Li W.H., Lin C.C., Shaw J.F., Wang S.Y. (2011). Metabolite profiles for *Antrodia cinnamomea* fruiting bodies harvested at different culture ages and from different wood substrates. J. Agric. Food Chem..

[5] Huang G.J., Deng J.S., Huang S.S., Lee C.Y., Hou W.C., Wang S.Y., Sung P.J., Kuo Y.H. (2013). Hepatoprotective effects of eburicoic acid and dehydroeburicoic acid from *Antrodia camphorata* in a mouse model of acute hepatic injury. Food Chem..

[6] Chen Y.C., Liu Y.L., Li F.Y., Chang C.I., Wang S.Y., Lee K.Y., Li S.L., Chen Y.P., Jinn T.R., Tzen J.T. (2011). Antcin A, a steroid-like compound from *Antrodia camphorata*, exerts anti-inflammatory effect via mimicking glucocorticoids. Acta Pharmacol. Sin..

[7] Hsieh Y.C., Rao Y.K., Whang-Peng J., Huang C.Y., Shyue S.K., Hsu S.L., Tzeng Y.M. (2011). Antcin B and its ester derivative from *Antrodia camphorata* induce apoptosis in hepatocellular carcinoma cells involves enhancing oxidative stress coincident with activation of intrinsic and extrinsic apoptotic pathway. J. Agric. Food Chem..

[8] Huo Y., Win S., Than T.A., Yin S., Ye M., Hu H., Kaplowitz N. (2017). Antcin H protects against acute liver injury through disruption of the interaction of c-Jun-N-terminal kinase with mitochondria. Antioxid. Redox Signal..

[9] Kuo Y.H., Lin C.H., Shih C.C. (2015). Antidiabetic and antihyperlipidemic properties of a triterpenoid compound, dehydroeburicoic acid, from *Antrodia camphorata* in vitro and in streptozotocin-induced mice. J. Agric. Food Chem..

[10] Lin C.H., Kuo Y.H., Shih C.C. (2017). Eburicoic acid, a triterpenoid compound from *Antrodia camphorata*, displays antidiabetic and antihyperlipidemic effects in palmitate treated C2C12 myotubes and in high-fat diet-fed mice. Int. J. Mol. Sci..

[11] Yeh C.T., Huang W.C., Rao Y.K., Ye M., Lee W.H., Wang L.S., Tzeng D.T., Wu C.H., Shieh Y.S., Huang C.Y. (2013). A sesquiterpene lactone antrocin from *Antrodia camphorata* negatively modulates JAK2/STAT3 signaling via microRNA let-7c and induces apoptosis in lung cancer cells. Carcinogenesis.

[12] Tikunov Y. (2005). A novel approach for nontargeted data analysis for metabolomics. Large-scale profiling of tomato fruit volatiles. Plant Physiol..

[13] Arbona V., Iglesias D.J., Talón M., Gómez-Cadenas A. (2009). Plant phenotype demarcation using nontargeted LC-MS and GC-MS metabolite profiling. J. Agric. Food Chem..

[14] Farag M.A., Mohsen M., Heinke R., Wessjohann L.A. (2014). Metabolomic fingerprints of 21 date palm fruit varieties from Egypt using UPLC/PDA/ESI–qTOF-MS and GC-MS analyzed by chemometrics. Food Res. Int..

[15] Cui Z.W., Xu S.Y., Sun D.M., Chen W. (2006). Dehydration of concentrated *Ganoderma lucidum* extraction by combined microwave vacuum and conventional vacuum drying. Dry. Technol..

[16] Dührkop K., Fleischauer M., Ludwig M., Aksenov A.A., Melnik A.V., Meusel M., Dorrestein P.C., Rousu J., Böcker S. (2019). SIRIUS 4: A rapid tool for turning tandem mass spectra into metabolite structure information. Nat. Methods.

[17] Lin Y.L., Lee Y.R., Tsao N.W., Wang S.Y., Shaw J.F., Chu F.H. (2015). Characterization of the 2,3-oxidosqualene cyclase gene from *Antrodia cinnamomea* and enhancement of cytotoxic triterpenoid compound production. J. Nat. Prod..

[18] CNS (2021). Fruiting Body of Niu-Chang-Chih (Ku).

[19] Chung C.H., Yeh S.C., Tseng H.C., Siu M.L., Lee K.T. (2016). Chemical quality evaluation of *Antrodia cinnamomea* fruiting bodies using phytomics similarity index analysis. J. Food Drug Anal..

[20] Liao S.C., Wen F.C., Chien W.C., Liao Y.H. (2017). Anticancer effects of different extracts of various *Antrodia cinnamomea* types on human liver HepG2 cells and analyses of compositions and bioactivities. Int. J. Adv. Sci. Eng..

[21] Liu S.C., Wu T.Y., Hsu T.H., Lai M.N., Wu Y.C., Ng L.T. (2022). Chemical composition and chronic toxicity of disc-cultured *Antrodia cinnamomea* fruiting bodies. Toxics.

[22] Zhang B.B., Guan Y.Y., Hu P.F., Chen L., Xu G.R., Liu L., Cheung P.C.K. (2019). Production of bioactive metabolites by submerged fermentation of the medicinal mushroom *Antrodia cinnamomea*: Recent advances and future development. Crit. Rev. Biotechnol..

[23] Zeng W.W., Chen T.C., Liu C.H., Wang S.Y., Shaw J.F., Chen Y.T. (2021). Identification and isolation of an intermediate metabolite with dual antioxidant and anti-proliferative activity present in the fungus *Antrodia cinnamomea* cultured on an alternative medium with *Cinnamomum kanehirai* leaf extract. Plants.

[24] Chiu H.H., Lee F.P., Wang J.K., Chou C.C. (2009). Identification and phylogenetic analysis of *Antrodia camphorata* and related species based on the polymorphic D2 region of LSU rDNA. Fooyin J. Health Sci..

[25] Li H.X., Wang J.J., Lu C.L., Gao Y.J., Gao L., Yang Z.Q. (2022). Review of bioactivity, isolation, and identification of active compounds from *Antrodia cinnamomea*. Bioengineering.

[26] Li G., Lou H.X. (2018). Strategies to diversify natural products for drug discovery. Med. Res. Rev..

[27] Chen W.L., Ho Y.P., Chou J.C. (2016). Phenologic variation of major triterpenoids in regular and white *Antrodia cinnamomea*. Bot. Stud..

[28] Ma T.W., Lai Y., Chen L.T., Yang F.C. (2016). The cultivation strategy of enhancing triterpenoid production in submerged cultures of *Antrodia cinnamomea* by adding monoterpenes. J. Taiwan Inst. Chem. Eng..

[29] Zhang Z., Wang Y., Yuan X.L., Luo Y.N., Luo M.N., Zheng Y. (2022). Effects of culture mechanism of *Cinnamomum kanehirae* and *C. camphora* on the expression of genes related to terpene biosynthesis in *Antrodia cinnamomea*. Mycobiology.

[30] Ma T.W., Lai Y., Yang F.C. (2014). Enhanced production of triterpenoid in submerged cultures of *Antrodia cinnamomea* with the addition of citrus peel extract. Bioprocess Biosyst. Eng..

[31] Shu C.H., Wu C.J., Hsiao W.J. (2015). Enhancement of triterpenoids production of *Antrodia cinnamomea* by co-culture with *Saccharomyces cerevisiae*. J. Bioprocess Biotech..

[32] Li H.X., Lu Z.M., Geng Y., Gong J.S., Zhang X.J., Shi J.S., Xu Z.H., Ma Y.H. (2015). Efficient production of bioactive metabolites from *Antrodia camphorata* ATCC 200183 by asexual reproduction-based repeated batch fermentation. Bioresour. Technol..

