# Supplementary material for: Metabolomic Profiling of Different Antrodia cinnamomea Phenotypes
Source: J Fungi (Basel). 2023 Jan 10;9(1):97. doi: 10.3390/jof9010097 (PMC9861778; doi:10.3390/jof9010097)
Supplement: Supplementary file 1 [file jof-09-00097-s001.zip › jof-2073340-supplementary resubmitted proof.pdf]

Supplementary Materials

Table S1. The phylogenetic identification of different *A. cinnamomea* (AC) phenotypes collected from the wild.

Sample	Location	ITS length (bps)	Similarity (%)	Accession number*
RACC	Chiayi	656	99.22	MN947413
RACN	Hualien	657	99.38	MN947414
RACF	Taitung	657	99.39	MK764936
RACTE	Chiayi	592	99.49	MN947415
WACF	Taitung	656	99.53	MK764938
WACD	Dongshi	658	99.08	MN947417
WACN	Hualien	657	99.38	MN947418
WACZ	Miaoli	654	99.69	MN947419
YACF	Tainan	656	99.23	MK764937
YACZ	Dongshi	657	99.38	MN947416

*Registered in the NCBI Genbank

RACC, RACN, RACF, and RACTE represent red AC (RAC) phenotypes

WACF, WACD, WACN, and WACZ represent white AC (WAC) phenotypes

YACF and YACZ represent yellow AC (YAC) phenotypes

Table S2. Compounds having significant contributions to each principal component [greater than 0.2; include principal component 1 (PC1) and principal component 2 (PC2)] in principal component analysis using the scaled relative peak areas (%) as independent variables.

Compound ID	HRMS (*m/z*)	t_R (min)	Identity	Loading	
				PC1	PC2
39	469.2956	6.25	25*R*-antcin C	0.47	0.26
38	469.2957	6.04	25*S*-antcin C	0.41	0.23
50	487.3061	4.00	25*S*-antcin K	0.31	0.07
18	425.3059	6.25	Unknown	0.26	0.14
17	425.3060	6.03	Unknown	0.24	0.12
87	409.3110	10.02	Unknown	0.21	0.13
101	935.5669	8.41	Unknown	-0.12	0.27
37	467.2800	8 41	25*S*-antcin B	-0.15	0.38
16	423.2902	8 41	Unknown	-0.35	0.68

Note: HRMS: the mass-to-charge ratio of $[M–H]^-$ measured by high-resolution mass spectrometry.

Table S3. Significantly different compounds of unidentified structure.

Compound ID	t_R (min)	HRMS (*m/z*)	Formula	Peak area (10000 units)		
				RAC	YAC	WAC
84	8.41	285.1495	$C_{18}H_{22}O_3$	168^a	103.4^b	53.7^c
3	8.41	407.2591	$C_{27}H_{36}O_3$	2098^a	991.4^b	822.2^b
4	4.00	407.2953	$C_{28}H_{40}O_2$	224^b	364^a	230.5^b
72	3.99	407.2953	$C_{28}H_{40}O_2$	220^b	402^a	249.3^b
73	7.12	409.2745	$C_{27}H_{38}O_3$	98^a	33^c	52.3^b
12	5.24	411.2903	$C_{27}H_{40}O_3$	127^b	164.5^a	95.5^c
119	4.87	413.3061	$C_{27}H_{42}O_3$	9^b	319.5^a	34.67^b
90	8.16	421.2746	$C_{28}H_{38}O_3$	544^a	160^b	114^b
16	8.41	423.2902	$C_{28}H_{40}O_3$	9678^a	4960^b	2798^c
113	6.59	423.2903	$C_{28}H_{40}O_3$	657^a	448^b	320^b
121	8.71	427.3217	$C_{28}H_{44}O_3$	12^b	297^a	7^b
120	8.69	427.3217	$C_{28}H_{44}O_3$	12^b	162^a	7^b
29	6.19	439.2851	$C_{28}H_{40}O_4$	443^a	191^b	148^b
122	8.74	439.2851	$C_{28}H_{40}O_4$	21^b	110^a	18^b
123	5.17	441.3007	$C_{28}H_{42}O_4$	611^a	322^b	281^b
30	5.23	441.3007	$C_{28}H_{42}O_4$	504^a	350^b	241^c
78	6.83	455.2800	$C_{28}H_{40}O_5$	462^a	172^b	159^b
95	4.22	455.2800	$C_{28}H_{40}O_5$	69^a	41^b	22^c
35	4.70	457.2956	$C_{28}H_{42}O_5$	416^b	727^a	321^b
124	4.89	457.2958	$C_{28}H_{42}O_5$	84^b	488^a	79^b
105	8.17	465.2645	$C_{29}H_{38}O_5$	233^a	82^b	62^b
104	8.17	465.2646	$C_{29}H_{38}O_5$	221^a	82^b	63^b
107	6.96	469.2959	$C_{29}H_{38}O_5$	444^a	282^b	150^c
106	6.93	469.2959	$C_{29}H_{38}O_5$	432^a	316^b	155^c
126	8.71	471.3116	$C_{29}H_{44}O_5$	21^b	462^a	11^b
125	8.69	471.3116	$C_{29}H_{44}O_5$	21^b	249^a	12^b
45	6.18	483.2747	$C_{29}H_{40}O_6$	1176^a	474^b	332^b
140	4.14	499.2696	$C_{29}H_{40}O_7$	514^a	119^b	99^b
63	6.82	501.2853	$C_{29}H_{42}O_7$	86^a	48^b	32^b
82	8.42	513.2855	$C_{30}H_{42}O_7$	476^a	376^b	214^c
99	5.24	517.3166	$C_{30}H_{46}O_7$	244^a	264^a	140^b

Note: RAC (red), YAC (yellow), and WAC (white) represent the color appearance of fruiting bodies of different *A. cinnamomea* phenotypes. Values are the average of four analyses (n = 4); different letters in the same column indicate significant differences between different AC phenotypes ($P < 0.05$; LSD test), and the difference in peak areas is represented by the depth of the gray-color shading. HRMS: the mass-to-charge ratio of $[M–H]^-$ measured by high-resolution mass spectrometry.
